# Evaluation of the peri-implant bone trabecular microstructure changes in short implants with fractal analysis

**DOI:** 10.1186/s40729-020-00209-7

**Published:** 2020-04-01

**Authors:** Hatice Cansu Kış, Ayşegül Güleryüz Gürbulak

**Affiliations:** 1 Department of Oral and Maxillofacial Radiology, Faculty of Dentistry, Nuh Naci Yazgan University, Kayseri, Turkey; 2 Department of Prosthetic Dentistry, Faculty of Dentistry, Erciyes University, Kayseri, Turkey

**Keywords:** Fractal analyses, Fractal dimension, Dental implants, Trabecular bone microstructure

## Abstract

**Backgrounds:**

This study aimed to evaluate the microstructural changes in the peri-implant bone in patients with short implants in terms of implant survival status by using fractal analysis measurements.

**Results:**

Dental panoramic radiographs (DPRs) of 67 patients were examined and included in this study. Fractal analysis and measurement of the crown-implant ratio were performed with ImageJ. The fractal analysis measurement was performed on the DPRs obtained at preoperative (FD0) and in the follow-up periods (after 2 ± 2 weeks (FD1), 2 months ± 2 weeks (FD2), 6 months ± 2 weeks (FD3), and 12 months + (FD4)). A *p* value < 0.05 was considered statistically significant. Power analyses were conducted for the test results that did not reject null hypothesis. A significant difference was found in the FD1 and FD2 values between the implant survival groups (*p* < 0.001 and *p* = 0.023, respectively). The mean FD1 and FD2 values of the success group were significantly higher than those of the failure group.

**Conclusions:**

Fractal analysis is a useful method to measure the trabecular microstructure of bone in non-standardized dental radiographs. The present study has a low power to reject the null hypothesis because of the low number of cases of implant failure. Therefore, further study with a large sample size is warranted. In clinical practice, the survival of implants may be predicted by analyzing fractal dimension of the surrounding trabecular bone of the implants.

## Backgrounds

Mandelbrot introduced fractals to describe his observation of shapes in nature, such as curves, surfaces, disconnected “dust,” and odd shapes. The word fractal originates from the Latin word “fractus,” which means broken. By using fractal mathematics, several studies have analyzed various fractal patterns in the human body. Fractal analysis is a mathematical method of describing complex shapes and structural patterns of the nature and is expressed numerically as fractal dimension [1, 2]. Trabeculae are thin columns with numerous large spaces that give a honeycomb or spongy appearance of cancellous bone, which is also called trabecular bone or spongy bone [3]. Previously, fractal analysis has been reported as a useful method to detect various diseases that affect the trabecular bone structure [4–6]. Especially in the field of dentistry, studies have indicated that fractal analysis by the box counting method can successfully evaluate trabecular changes in the mandible of patients with osteoporosis [7–9] and periodontal diseases [10] and in lactating women [11]. Furthermore, studies have been conducted to determine the changes in trabecular bone induced by surrounding bone tissues of implants [12–15].

Many studies have examined the effect of fractal analysis on image acquisition parameters [16, 17]. Although some studies have stated that fractal measurement of trabecular bone microstructures is affected by exposure time and noise, most studies support the belief that fractal measurement is relatively affected by imaging parameters but do not lead to a significant difference [18, 19]. One study that investigated the diagnostic imaging of the trabecular bone structure of oral implants by using cone beam computed tomography (CBCT) reported the highest accuracy of the measurement of fractal analysis. This study also highlighted the disadvantage of CBCT with regard to its accessibility and cost in dental clinical practice; however, it emphasized the practicality and accessibility of panoramic and periapical dental radiographs [20]. Fractal analysis can be performed on nonstandardized dental radiographs to assess pathological changes in bone or to assess the quality of peri-implant bone [17, 19, 21].

The quality of bone tissue at the site of implantation can be determined preoperatively with high accuracy, and changes in the trabecular structure, which is vital for the primary and secondary stability of the implant, can be observed during the follow-up after implantation.

Previous studies have evaluated fractal analysis of peri-implant bone before and after loading. However, no study has examined fractal analysis of the surrounding bone tissue of short implants or the changes in fractal analysis values of peri-implant bones according to jaws and restorative properties.

The present study aimed to evaluate the microstructural changes in the peri-implant bone in patients with short implants in terms of the implant survival status by using fractal analysis measurements. If the fractal analysis measures trabecular bone microstructural changes successfully, then the fractal dimensional changes of the peri-implant bones of the implants that failed will be significantly different.

## Materials and methods

### Study participants

**Fig. 1:**
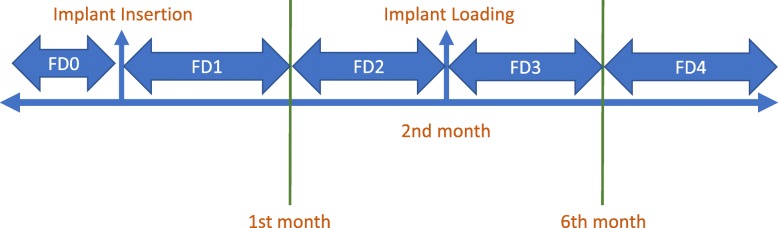
Fractal dimension values measured from the same area of interest on each panoramic radiograph over five different time intervals are shown in the figure. FD0, fractal dimension 0 (preoperative); FD1, fractal dimension 1 (0–1 months of follow-up); FD2, fractal dimension 2 (1–3 months of follow-up); FD3, fractal dimension 3 (6–12 months of follow-up); FD4, fractal dimension 4 (12 + months of follow-up)

This retrospective study was conducted in the dental clinic of Oral and Maxillofacial Radiology department and was approved by the local ethics committee (2013/203). The participants had approached the Prosthodontics Clinic between 2012 and 2019 for partial or complete tooth complaints. Among the data of 116 patients reviewed, panoramic radiographs of 67 patients were examined and included in this study. The panoramic radiographs were selected according to the following inclusion criteria: (1) no apparent observations of excessive imaging artifact; (2) patients who had all five panoramic radiographs—before implantation, immediately after prosthodontic loading (2 ± 2 weeks), at 2 months ± 2 weeks after implantation, at 6 months ± 2 weeks after loading, and at 12 months or more after loading (Fig. 1.); and (3) panoramic radiographs of patients without bone metabolism disease. Informed consent was obtained from all patients before their enrollment in the study. Information about the medical status of the patients was obtained from their anamnesis records.

### Radiographic technique

**Fig. 2:**
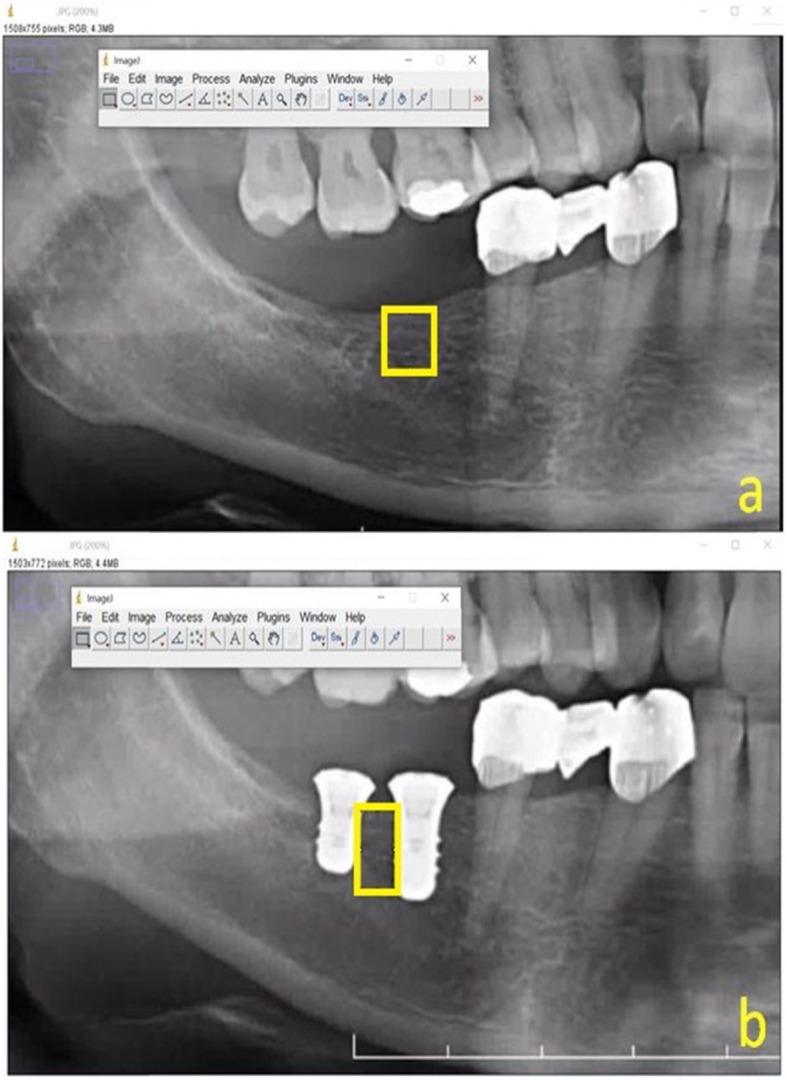
Region of interests (ROIs) were selected arbitrarily in **a** preoperative radiographic image and **b** a follow-up radiographic image

Sixty-seven dental panoramic radiographs (DPRs) were evaluated. All DPRs were obtained with the same radiography device (OP200 D; Instrumentarium Dental, Tuusula, Finland; radiography parameters, 66–85 kVp, 10–16 mA, 14.1 s exposure time). Patients were positioned for radiography according to the manufacturer’s recommendations; the Frankfort horizontal plane was parallel to the floor and the sagittal plane was aligned with the vertical line of the device. The region of interest (ROI) was arbitrarily selected on each radiograph (Fig. 2).

### Fractal dimension calculation

**Fig. 3:**
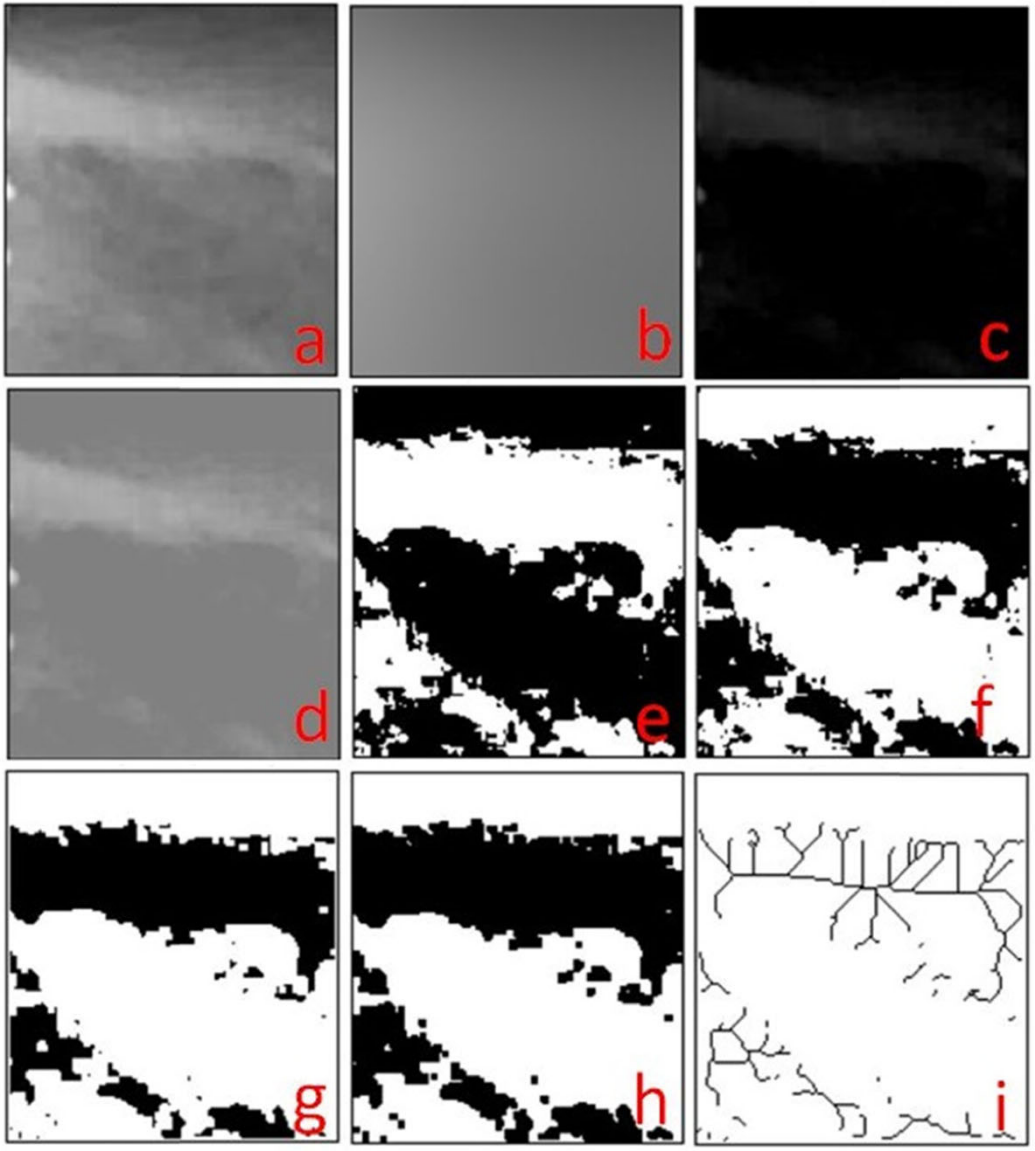
Fractal analysis stages. **a** Selected region of interest (ROI). **b** Cropped and duplicated version of ROI. **c** Addition of Gaussian filter. **d** Subtraction. **e** Addition of 128 pixels. **f** Binarized version. **g** Eroded version. **h** Dilated version. **i** Inverted version **j** Skeletonization

Fractal analysis was performed using the box counting method developed by White and Rudolph [22] and Geraets et al. [23]. The DPRs were analyzed with the ImageJ version 1.38x software (National Institute of Health, Bethesda, MD, USA) on a Dell Precision T5400 workstation (Dell, TX, USA) with a 32-inch Dell liquid crystal display screen with a resolution of 1280 × 1024 pixels in a darkroom. After selection of ROI, the image was duplicated (Fig. 3a). The image was then blurred with a Gaussian filter. Overlapping soft tissues or ghost images of the anatomical structures were removed with the density correction step of the Gaussian filter with large-scale alterations in image brightness caused by varying thickness of the object. Only large differences in density were retained (Fig. 3b). The generated blurred images were subtracted from the original image (Fig. 3c). A gray value of 128 was added to each pixel location, which resulted in an image with individual alterations that reflect certain properties with different brightness, such as the trabeculae and bone marrow (Fig. 3d). A binary image was generated by thresholding with a brightness value of 128. In this process, the image was segmented into regions that represented the bone marrow and trabeculae (Fig. 3e). Thereafter, the image was inverted, and the segments that represented the trabeculae were set to black color, and the bone marrow was set to white color (Fig. 3f). The resulting eroded and dilated image had reduced noise (Fig. 3 g, h). Lastly, with the skeletonization process, the image was further eroded until only the central line of pixels remained (Fig. 3i). The box counting algorithm provided by the software was used for fractal analysis of the reduced images. The image was divided into squares of pixels of size 2, 3, 4, 6, 8, 12, 16, 32, and 64. The squares that included the segments of trabeculae and the total number of squares were calculated for each pixel size. A logarithmic scale graph of the obtained values was plotted. The dimensional value was obtained from the slope of the line that was drawn according to the plotted points on the logarithmic graph.

### Measurement of the crown-implant ratio

**Fig. 4:**
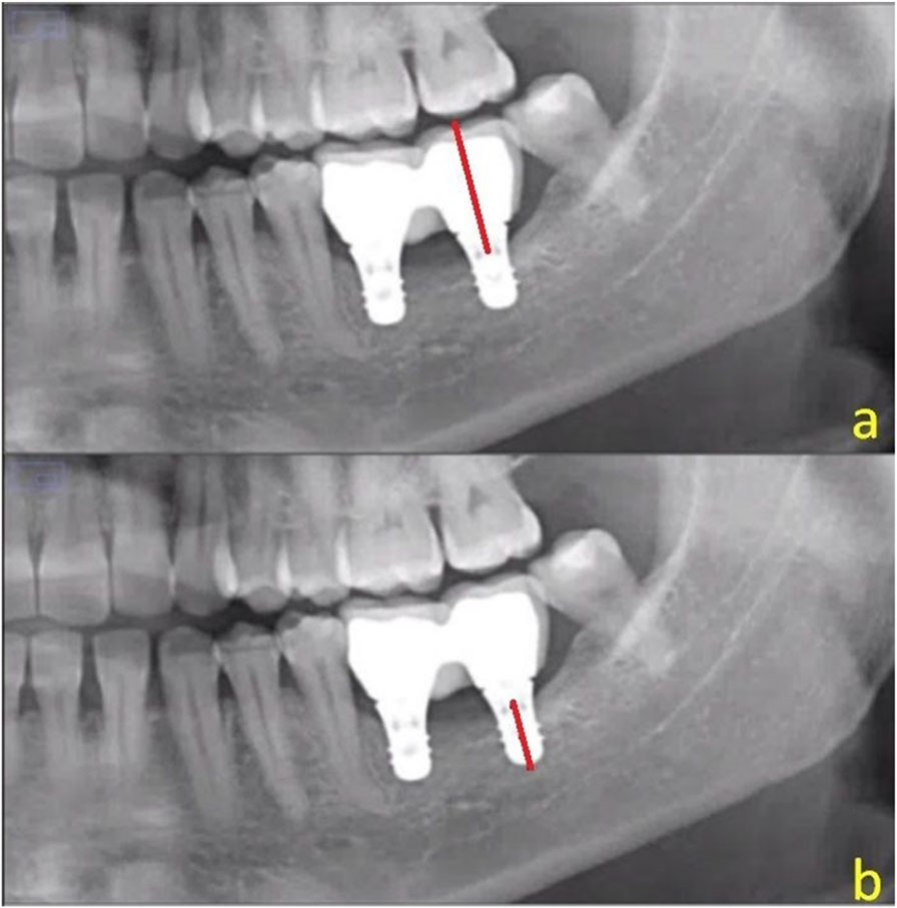
The crown-implant ratio measurement showing **a** the length of the crown (red line) and **b** the length of the implant (red line)

The crown-implant ratio was measured using the ImageJ version 1.38 software measuring tool in conjunction with a magnification tool. Each implant was measured from its bottom to the crown base and then from the crown base to its highest point (Fig. 4).

All measurements were performed by a dento-maxillofacial radiologist who was blinded to patient information. To evaluate the intra-observer correlation, 20% of the images were randomly selected, and fractal analysis measurements were performed by the same radiologist 1 month later.

### Statistical analyses

Statistical analyses were conducted with the R Statistical Software version 3.0.2 (Foundation for Statistical Computing, Vienna, Austria) and TURCOSA (Turcosa Analytics Ltd. Co., Turkey). Normal distribution of the data was evaluated with the Shapiro–Wilk test. Pearson’s correlation coefficient analysis was used to evaluate the inter-observer correlations and for comparison of numeric variables. Homogeneity of variances was evaluated with Levene test. Kruskal–Wallis test, Mann–Whitney *U* test, Student’s *t* test, and Welch two-sample *t* test were performed to compare the numerical variables between the categorical between the successful and failed implant groups. Fisher’s exact test was used to compare categoric variables between the implant survival groups. A probability level of less than 5% (*p* < 0.05) was accepted as statistically significant. Power analyses were conducted for the test results that did not reject null hypothesis.

## Results

**Fig. 5:**
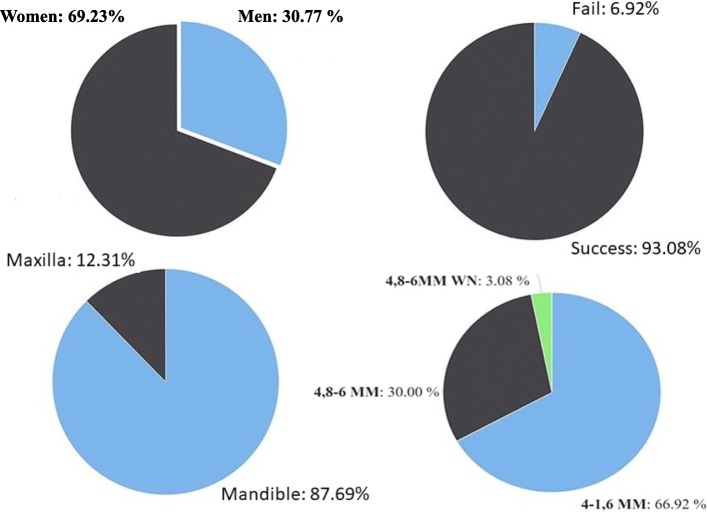
Pie charts shows the distribution of the demographic datas of the patients

**Fig. 6:**
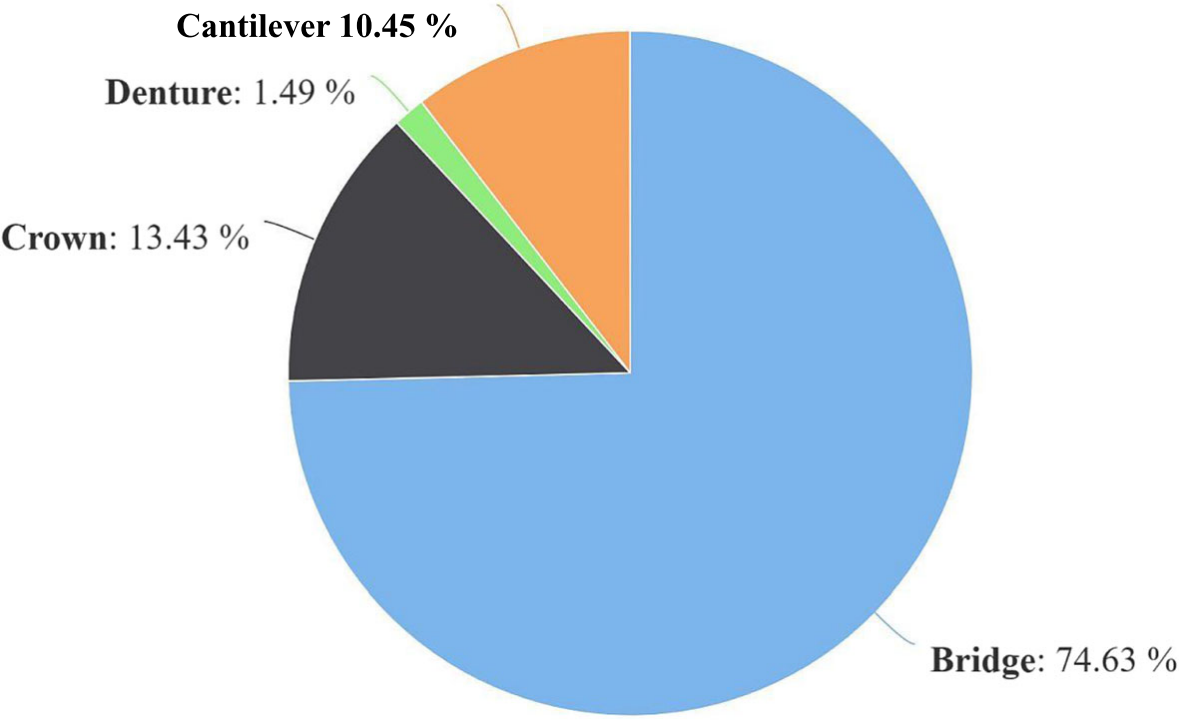
Pie chart shows the distribution of loaded implants prosthetic restorations

**Fig. 7:**
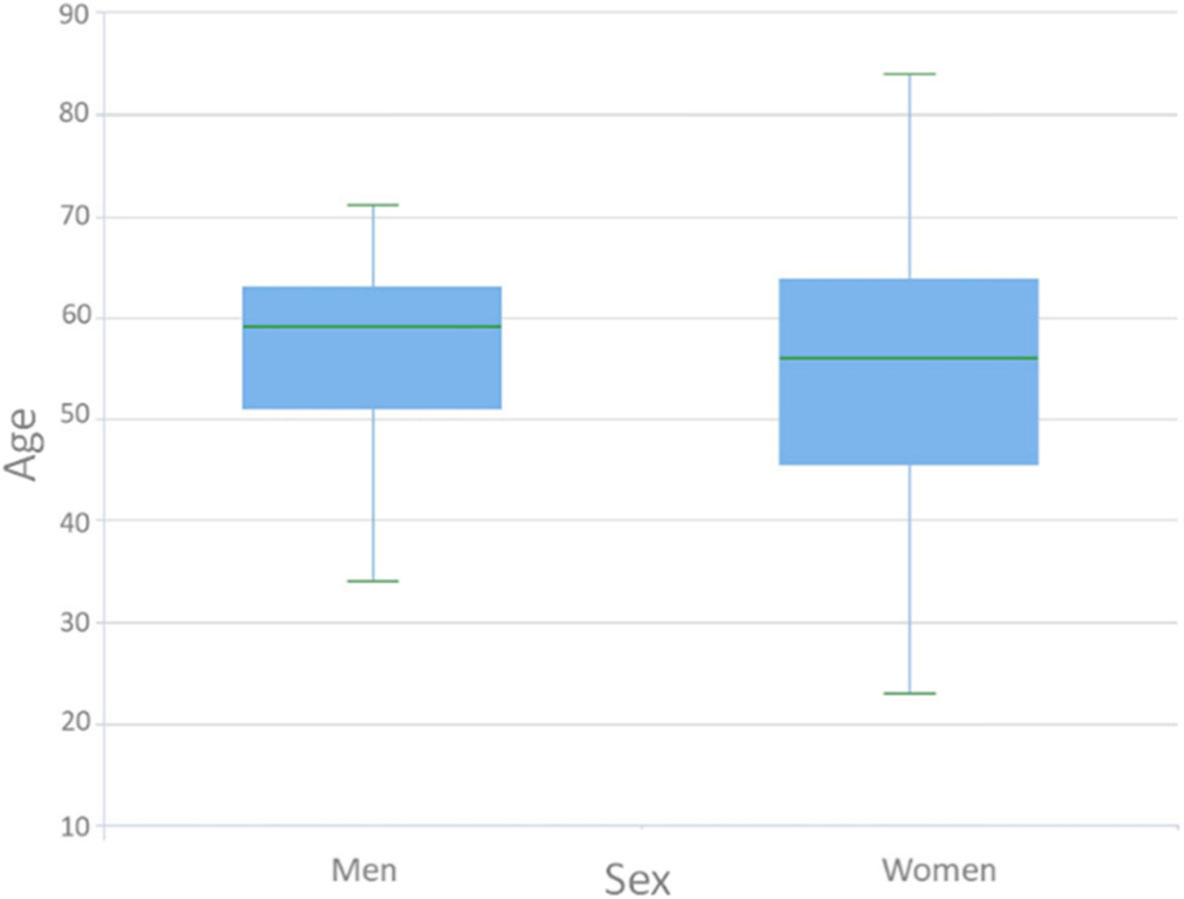
The box plot shows the distribution of age between the sex groups

Descriptive statistics were performed. The data were not normally distributed (*p* < 0.05). The intra-observer correlation coefficients of repeated measurements were 0.927, 0.889, 0.913, 0.988, 0.961, and 0.936 for FD0 (fractal dimension), FD1, FD2, FD3, FD4, and crown-implant ratio, respectively. Descriptive data are shown in Figs. 5, 6, and 7. A significant difference was found for sex between the implant survival groups (*p* = 0.024). There was no significant difference between the implant survival groups for patients’ age, type of implant, jaw of the inserted implant, and FD0 (preoperative fractal dimension values). Moreover, there was no significant difference between the implant survival groups for crown-implant ratios measured after implant loading (*p* = 0.101). Table 1 shows the distribution and description of FD1 and FD2 values, crown-implant ratios, sex difference between the successful and failed implant survival groups, and power and effect size of the tests. A significant difference was found in the FD1 and FD2 values between the implant survival groups (*p* < 0.001 and *p* = 0.023, respectively). The mean FD1 and FD2 values of the success group were significantly higher than those of the failure group. The mean FD values before and after implant insertion and loading showed no significant difference (*p* > 0.05) (Table 2.). There was no significant correlation between FD3 and FD4 values and crown-implant ratios measured after implant loading (*p* > 0.05). Furthermore, there was no significant difference in the FD3 and FD4 values between the groups for different types of prosthetic restorations (*p* > 0.05).

**Table 1:** Distribution and description of FD1 and FD2 values, crown-implant ratios, and sex difference between the implant failure and success groups

		Failure (*n*)	Success (*n*)	*P* value	Power	Effect size
Sex	Women	3	87	0.024	0.68	0.21
	Men	6	34			
FD1		9 0.82 ± 0.28 (mean) 0.45 (min)–1.26 (max)	121 1.13 ± 0.25 (mean) 0.41 (min)–1.51 (max)	< 0.001	0.99	1.45
FD2		9 0.97 ± 0.24 (mean) 0.61 (min)–1.36 (max)	121 1.13 ± 0.19 (mean) 0.41 (min)–1.51 (max)	0.023	0.99	0.79
Crown-implant Ratio		2 6.51 ± 3.89 (mean) 3.77 (min)–9.27 (max)	65 4.61 ± 1.58 (mean) 2.57 min)–10.67 (max)	0.101	0.99	1.19

*FD1* fractal dimension 1 (0–1 months of follow-up), *FD2* fractal dimension 2 (1–3 months of follow-up)

**Table 2:** Mean fractal dimension (FD) values before and after implant insertion

	*n*	Mean	Standard deviation	Minimum	Maximum
FD0	130	1.243	0.152	0.750	1.560
FD1	130	1.113	0.224	0.405	1.510
FD2	130	1.116	0.196	0.410	1.510
FD3	67	1.092	0.216	0.430	1.500
FD4	67	1.081	0.247	0.430	1.500

*FD0* fractal dimension 0 (preoperative), *FD1* fractal dimension 1 (0–1 month of follow-up), *FD2* fractal dimension 2 (1–3 months of follow-up), *FD3* fractal dimension 3 (6–12 months of follow-up), *FD4* fractal dimension 4 (12 + months of follow-up)

## Discussion

This study aimed to evaluate the microstructural changes in the peri-implant bone in patients with short implants in terms of the implant survival status by using fractal analysis measurements.

In this study, a significant difference was found in the FD1 and FD2 values between the implant survival groups, and the mean FD1 and FD2 values of the success group were significantly higher than those of the failure group. This result indicates that the assessment of fractal analysis at 3 months after implant insertion may be useful to determine the probability of cases of implant failure. According to our results, the fractal analysis values of the peri-implant bones of cases of implant failure were significantly lower at 0–1 month (FD1) and 1–2 months (FD2) after implantation. This may have contributed to the reduction of trabecular bone density in the bone around the implantation site.

In our study, the ratio of crown-implant length showed no statistically significant difference compared to the success rate of implantation for post-loading implants; however, the small sample size may have contributed to this result. In addition, the values of mean crown-implant ratio were higher in the failure group than in the success group; this finding could be interpreted as an indicator of clinical significance. There was no clinically significant correlation between the crown-implant ratio and FD3 and FD4 values measured after implant loading.

Some studies have stated that measurements of fractal analysis are affected by image noise and exposure parameters, and therefore, these analyses should be applied to standardized radiographs [16, 24]. In contrast, other reports indicate that image acquisition and exposure parameters do not significantly affect measurements of fractal analysis [14, 15, 19, 20].

Ibrahim et al. [20] performed fractal analysis measurements with CBCT and demonstrated high accuracy of measurements as compared to dental radiographs for the diagnosis and follow-up of implant. However, the effective exposure dose during dental tomography is considerably higher than that during dental radiography. Therefore, CBCT is not indicated for the assessment of implant follow-up for all cases of implantation [25].

Fractal analysis of bone microstructure on dental radiographs may be useful for diagnostic applications; however, the histological microstructures of the bone cannot be visualized by any clinical imaging modality. Corpas et al. [12] stated that minor changes in bone occurring over a short-term period can be followed up with digital intraoral radiography; however, the results of radiographic fractal analysis did not match that of histological fractal analysis.

The box counting method quantitatively describes the severity of bone disease and can be used to improve the current diagnostic techniques. Updike et al. [10] found that the fractal analysis determined the differences between the bones affected and not affected by periodontal diseases.

Coşgunarslan et al. [11] evaluated 240 DPRs of lactating (3–6 months duration) and nulliparous women by the fractal box counting method and found a significant difference between the FD values of the cancellous bone but no significant difference between the FD values of the cortical bones. This observation may have resulted from the fact that fractals affect cortical bone much later than the cancellous bone. Further study is needed to assess fractal analysis of the cortical bones.

Fractal dimension values on dental radiographs have been reported to differ between dentate and edentulous patients [19]. Moreover, the quality of trabecular bone architecture can be determined with fractal analysis on direct digital dental radiographs.

Zeytinoğlu et al. [15] reported significantly reduced mean FD values of the peri-implant trabecular bone at 6 months after prosthetic loading. Contrastingly, Mu et al. [14] found significant increase in the mean fractal dimension at 12 months after prosthetic loading. According to our results, the mean fractal dimension values decreased at 6–12 months after implant loading, but no significant difference was found.

There are some limitations of this study. One of them is the limited sample number of the failed implants. To assess the required sample size, power analyses were conducted. Unfortunately, we have limited number of follow-up radiographs of patients with failed implants. This study may be useful as a pilot study for further studies with much more sample size. Second, although periapical radiography is a high-resolution intraoral imaging method for FD analysis, panoramic radiographs were used because this study was retrospective. Finally, in this study, the ROI selection was not a specific frame size. The effect of ROI position and size on FD measurements is unclear. It has been proved that determining the exact ROI location and size may not make a significant difference, but there is no consensus on this idea [26].

## Conclusion

Fractal analysis is a useful method to measure the trabecular microstructure of bone in nonstandardized dental radiographs. The present study has a low power to reject the null hypothesis because of the low number of cases of failed implants. Therefore, further studies with a large sample size are warranted. Assessing a series of studies can provide certain cut-off values; this can enable to routinely use fractal analysis to assess bone quality on radiological images before implantation in clinical settings.

## Data Availability

Not Applicable

## Abbreviations

DPRs: Dental panoramic radiographs
FD0: Fractal dimension 0 (preoperative)
FD1: Fractal dimension 1 (0–1 months of follow-up)
FD2: Fractal dimension 2 (1–3 months of follow-up)
FD3: Fractal dimension 3 (6–12 months of follow-up)
FD4: Fractal dimension 4 (12 + months of follow-up)
CBCT: Cone beam computed tomography
ROI: Region of interest
